# Efficacy and safety of oral robenacoxib (tablet) for the treatment of pain associated with soft tissue surgery in client-owned dogs

**DOI:** 10.1186/s12917-017-1100-x

**Published:** 2017-06-26

**Authors:** Gabriele Friton, Caryn Marie Thompson, Daniela Karadzovska, Stephen King, Jonathan N. King

**Affiliations:** 1 Elanco Animal Health Inc., Companion Animal Development, CH-4058 Basel, Switzerland; 2 Elanco Animal Health Inc., Companion Animal Development, Greenfield, IN 46140 USA; 3 Elanco Australasia Pty Limited, Food Animal Development, Kemps Creek, NSW 2178 Australia

**Keywords:** Analgesia, Dog, NSAID, Robenacoxib, Peri-operative, Soft tissue surgery

## Abstract

**Background:**

Non-steroidal anti-inflammatory drugs (NSAIDs) have been proven to be effective in controlling peri-operative pain in dogs. Robenacoxib is an NSAID with high selectivity for the cyclooxygenase (COX)-2 isoform. The objective of this study was to assess the efficacy and safety of an oral tablet formulation of robenacoxib in client-owned dogs undergoing soft tissue surgery. The study was a prospective, multi-center, randomized, masked, placebo-controlled, parallel-group clinical trial. A total of 239 dogs were included and randomly allocated in a 1:1 ratio to receive either robenacoxib or placebo. Each dog received an oral tablet administration of either robenacoxib, at a target dose of 2 mg/kg, or placebo once prior to surgery and for two additional days post-operatively. All dogs also received a pre-anesthetic dose of 0.2 mg/kg butorphanol (intravenous or intramuscular). Pain assessments were performed using the short form of the Glasgow Composite Measure Pain Scale. Robenacoxib was compared to the placebo group on a success/failure basis. Treatment failure was defined as the need for rescue therapy to control post-operative pain.

**Results:**

Significantly (*P* = 0.019) more dogs administered robenacoxib were considered treatment successes (89 of 116, 76.72%) compared to dogs given placebo (74 of 115, 64.35%). The percentage of treatment failure was therefore 23.28% in the robenacoxib and 35.65% in the placebo group. The least squares mean total pain scores were significantly different between groups and in favor of robenacoxib at 3 and 5 hours (*P* < 0.05) and 8 hours post-extubation (*P* < 0.01). Pain at the surgery sites (response to touch) was also significantly improved at 3, 5 and 8 hours post-extubation in dogs receiving robenacoxib versus placebo (*P* < 0.01). In addition, a significant overall improvement in posture/activity was revealed with robenacoxib having lower scores versus placebo (*P* < 0.01). No significant differences between the robenacoxib and placebo groups in the frequency of reported adverse events were observed.

**Conclusions:**

Robenacoxib by oral (tablet) administration was effective and well tolerated in the control of peri-operative pain and inflammation associated with soft tissue surgery in dogs.

## Background

Non-steroidal anti-inflammatory drugs (NSAIDs) have been proven to be effective in controlling post-operative pain in dogs when used either alone or in combination with opioids [1–3]. Robenacoxib is an NSAID which produces highly selective inhibition of the cyclooxygenase (COX)-2 isoform of COX, and significantly inhibits COX-2 and spares COX-1 in vivo when administered orally at the recommended dosages [4–6]. Robenacoxib has a fast onset of action, achieves maximal blood concentrations within 0.25–0.5 h after both oral and subcutaneous dosing, and has a high bioavailability (84% oral; 88% subcutaneous) [7]. The dose and concentration-effect relationships for single oral doses of robenacoxib over the dose range 0.5–8.0 mg/kg were established in a urate crystal-induced acute synovitis model in dogs; robenacoxib increased the weight-bearing and decreased pain and swelling [6]*.*

The safety of robenacoxib was investigated in the dog in two randomized, placebo-controlled, parallel-group studies. Robenacoxib was administered orally once daily to healthy young beagle dogs at 0 (placebo), 10, 20 and 40 mg/kg for 1 month (Study 1) and at 0 (placebo), 2, 4, 6 and 10 mg/kg for 6 months (Study 2) [8]. Relative to placebo, no significant adverse effects of robenacoxib were recorded in either study for clinical observations, clinical chemistry and hematological variables, and macroscopic or microscopic lesions at necropsy. In Study 2, additional examinations identified no adverse effects of robenacoxib on buccal bleeding time, electrocardiographic and ophthalmoscopic examinations, urinalysis or stifle joint tissues. The lack of adverse events (AEs) at doses as high as 40 mg/kg versus recommended doses ranging from 1 to 4 mg/kg indicates a high safety index of robenacoxib in dogs, attributed to a combination of its high COX-2 selectivity and short residence time in the central compartment [8–10].

In view of its pharmacological properties, robenacoxib has the potential to provide optimal peri-operative pain control in dogs. Previous studies investigated robenacoxib for the management of peri-operative pain and inflammation in dogs undergoing various surgeries [11, 12]. Robenacoxib has been approved for the control of pain and inflammation associated with soft tissue surgery in many countries (e.g. http://www.ema.europa.eu/ema/) [13]. The most recent registration was in the USA (https://www.fda.gov/) [14], which was supported by the results of the study described in this manuscript.

The objective of this study was to demonstrate the field effectiveness and safety of robenacoxib tablets, administered orally at a dose of 2 mg/kg (an inherent dose band of 2–4 mg/kg) once daily for three days, for the control of post-operative pain and inflammation associated with soft tissue surgery in dogs. We hypothesized that robenacoxib administered prior to surgery and for two additional days post-operatively would have superior analgesic effects when compared to a placebo. Efficacy was measured by success/failure criteria based on pain assessments and the need for rescue therapy.

## Methods

### Study design

The study was a prospective, multi-center, randomized, masked, placebo-controlled, parallel-group clinical trial at 11 companion animal veterinary clinics located at various geographic locations within the USA. The study was conducted in accordance with guidelines for Good Clinical Practice (VICH GL9), Adequate and Well-controlled Studies (21 CFR 514.117), and New Animal Drugs for Investigational Use (21 CFR 511.1).

The protocol was reviewed and approved by the Sponsor’s Institutional Animal Care and Use Committee. All owners provided written consent at the pre-enrollment visit (Day −14 to −2) for their dog to enter the study. This manuscript was prepared after consideration of the CONSORT guidelines on randomized trials [15].

### Selection criteria

The inclusion criteria comprised dogs aged ≥6 months, either sex, any breed, weighing at least 2.5 kg at the time of enrollment and scheduled to undergo soft tissue surgery (e.g. ovariohysterectomy, cryptorchidectomy, splenectomy, cystotomy, or major external surgeries, such as mastectomy or skin tumor removal (mass ≥ 8 cm in size)). Aside from needing soft tissue surgery, dogs were clinically healthy and had acceptable clinical pathology results as determined by the Investigator. Dogs meeting any of the following exclusion criteria were not enrolled in the study: those that had a known hypersensitivity to NSAIDs or sulfonamide drugs; were being used for breeding, or were pregnant or lactating; were receiving anticonvulsant, behavioral or cardiac medications; were dehydrated or were receiving concomitant diuretic therapy; had existing cardiovascular, gastrointestinal tract, hepatic and/or renal dysfunction; had uncontrolled endocrine or systemic disorders such as diabetes mellitus, hypothyroidism, or other systemic disorders (dogs requiring treatment for diabetes mellitus or hypothyroidism had to be stabilized for at least 28 days prior to enrollment; stable status was documented by clinical pathology); within 14 days prior to enrollment had undergone invasive surgical procedures or procedures that would interfere with an accurate assessment of pain; had a concurrent painful condition other than the presenting condition, which could have interfered with pain assessments; had been treated with topical or systemic anti-inflammatory products such as NSAIDs within 14 days prior to enrollment, short-acting (systemic or local) corticosteroids within 30 days prior to enrollment or long-acting corticosteroids within 60 days prior to enrollment; had been treated with anesthetics, sedatives, tramadol or tranquilizers within 2 days prior to enrollment; exhibited aggressive or frightened behavior that would cause difficulty in clinical examinations, collection of clinical specimens or administration of treatments; had a known intolerance to the anesthetics used in the study or had received alternative forms of pain relief (e.g. acupressure, acupuncture, chiropractic manipulation, clinical therapy) within 14 days prior to enrollment. Dogs belonging to an employee of the sponsoring company or other animal health drug manufacturer, an Investigator or the Food and Drug Administration were also not eligible for enrollment in the study.

Dogs meeting any of the following criteria after inclusion were withdrawn from the study: those that required pain intervention with a Glasgow Composite Measure Pain Scale - Short Form (CMPS-SF) score of ≥6 (considered a treatment failure); exhibited an AE that compromised their ongoing treatment or the integrity of the study; were fractious and unable to continue in the study; received forbidden concomitant treatment; were affected by protocol deviation(s) that compromised the integrity of the study or a disorder that could have interfered with the evaluation of their response to treatment, or for any other reason as determined by the Investigator in consultation with the Sponsor. Owners or Investigators could also decide to withdraw the dog for efficacy or safety reasons and the study could have been stopped by the Sponsor at any time point if required.

### Anesthesia and analgesia protocol

All dogs were adequately hydrated prior to and during surgery. As an anesthetic pre-medication, all dogs received intravenous or intramuscular administration of butorphanol at a dose of 0.2 mg/kg body weight after dosing with robenacoxib or placebo approximately 45 min prior to surgery. Agents including propofol, thiopental, sevoflurane and isoflurane were allowed to facilitate induction, maintenance and recovery from anesthesia. Local anesthesia was not permitted for any dog.

### Randomization and treatment

Dogs were formally included on Day 0 and allocated randomly to treatment groups in a 1:1 ratio in blocks of four in order of enrollment. Dogs were administered either the oral (tablet) formulation of robenacoxib (Onsior®, Elanco Animal Health, Greenfield, USA) or placebo tablet (Elanco France, Huningue, France) once daily for 3 days. Robenacoxib was administered orally, as whole tablets (10 mg, 20 mg and 40 mg), at a target dose of 2 mg/kg of body weight (an inherent dose band of 2 to 4 mg/kg) once daily for three consecutive days. For the first dose, food was withheld overnight. For the second and third doses, robenacoxib was administered without food or with a small amount of food. The dose administered to each dog was calculated from the pre-anesthetic body weight measured at Day −1 or Day 0. The first treatment was given approximately 45 min prior to surgery at the time of pre-anesthetic medication. Subsequent once daily treatments were given at approximately the same time each day. Re-dosing was performed if the dog vomited within 5 min of administration and the tablet was recognizable in the vomitus. If the dog vomited and the tablet was not recognizable, the dog was not re-dosed. The administration methods for the placebo tablet were the same as those described for robenacoxib tablets.

The randomization list was computer-generated by the statistician using SAS/STAT® software (Version 9, SAS Institute Inc., Cary, NC, USA). Blinding was accomplished by the identical appearance of the tablets (same formulation except that the placebo tablets had no active ingredient) and packaging in both groups, and in addition by separation of functions: a treatment administrator (i.e. dispenser) at each clinic was responsible for dispensation and administration of test items and reconciliation of used and unused products. All study site personnel were masked to treatment assignment except the dispenser.

### Clinical examination and follow-up

Clinical examinations were performed at enrollment, at scheduled study completion, in cases of early withdrawal, and for any animal which experienced a serious AE. The examination included a routine assessment of general appearance, major systems and body weight. A description of scheduled study activities is depicted in Table 1.

**Table 1:** Schedule of study activities

Study Days	Activity
Day −14 to −2	Pre-enrollment visit
	Blood and urine sample collection
Prior to enrollment	Review clinical pathology results for animal eligibility
Day −1 to 0	Physical examination
	Body weight determination
	Begin acclimatization at least 2 h before performing the baseline CMPS-SF assessment
Day 0	Baseline pain assessment using CMPS-SF
	First treatment
	Administration of butorphanol
	Induction of anesthesia, perform soft tissue surgery, extubation
	Pain assessment using CMPS-SF
	- 1.5, 3, 5 and 8 h (± 30 min) post-extubation
	- between scheduled assessments, if pain intervention required^a^
Day 1	Pain assessment using CMPS-SF 24 h (± 1 h) after initial treatment and prior to second treatment
	Second treatment
	Pain assessment using CMPS-SF
	- 2 h and 8 h (± 30 min) post-treatment
	- between scheduled assessments, if pain intervention required^a^
Day 2	Pain assessment using CMPS-SF 48 h (± 1 h) after initial treatment and prior to third treatment
	Third treatment
	Pain assessment using CMPS-SF
	- 2 h and 4 h (± 30 min) post treatment - between scheduled assessments, if pain intervention required^a^
	- if pain intervention not required within 4 h (± 30 min) post treatment: - Exit physical examination - Body weight determination - Blood and urine sample collection
Day 3 to 10 after study exit	Post-study follow up phone call

*CMPS-SF* Glasgow Composite Measure Pain Scale - Short Form, *h* hours, *min* minutes

^a^if pain intervention required: administer intervention treatment, exit physical examination, determine body weight, collect blood and urine sample, remove dog from the study and monitor in clinic for 24 h

### Surgical procedures

Surgery start time was defined as the time of first (skin) incision. If surgery start time was delayed and was ≥60 min after the first dose of robenacoxib or placebo administration, the Investigator was instructed to stop study procedures, and observe at least a 2-day washout period prior to re-dosing and surgery.

### Rescue therapy

Intervention treatment (“rescue therapy”) was administered at any time the Investigator determined that a dog was excessively uncomfortable or in pain, and/or a score of ≥6 was determined for the CMPS-SF during pain assessment. Intervention treatment could include any product (except other NSAIDs or corticosteroids) used to control pain.

### Premature completion and follow-up

Dogs could be withdrawn from the study and/or receive rescue therapy at any time at the discretion of the veterinarian. Dogs receiving intervention treatment were observed in the clinic for a minimum of 24 h post-intervention and any potential AEs were documented. The owners of study dogs received a follow-up phone call approximately 3 to 10 days after normal or premature completion to assess the animal’s general well-being.

### Efficacy assessments

The primary outcome variable was treatment failure, which was defined as the need for rescue therapy to control post-operative pain or premature withdraw of the dog from the study due to an AE which was considered possibly or probably related to treatment. Need for rescue therapy was decided by the Investigator based on either a score of ≥6 on the CMPS-SF [16] (Appendix) or if the Investigator determined at any time that rescue (pain) therapy was needed. Robenacoxib was compared to the placebo group on a success/failure basis. Investigators were instructed that the same clinician should make all efficacy assessments for all cases at each site.

Secondary outcome variables included the total CMPS-SF score and the six components of the CMPS-SF (vocalization, attention to wound area, mobility, response to touch, demeanor and posture/activity). A categorical score was assigned within each behavior category based on the severity of the behavior or response by the dog.

A baseline evaluation was performed on Day 0 after the dog had acclimatized for at least 2 h in the clinic, and prior to administration of the test items and pre-anesthetic agents. Thereafter evaluations for the primary and secondary outcome variables were conducted: on Day 0 post-surgical extubation at 1.5, 3, 5 and 8 h (±30 min); on Day 1 at 24 h (±1 h) after initial administration and prior to second treatment, and thereafter at 2 and 8 h (±30 min); on Day 2 at 48 h (±1 h) after initial administration and prior to third treatment, and thereafter at 2 and 4 h (±30 min).

### Safety assessments

Safety was analyzed in all dogs that had received at least one dose of robenacoxib or placebo, from reported AEs, post-study owner follow-up findings, clinical pathology variables (hematology, serum chemistry and urinalysis) and changes in body weight.

### Statistical analysis

The study was planned to include a minimum of 220 dogs, with 110 dogs from each group receiving either robenacoxib or placebo. All analyses were performed using SAS/STAT® software (Version 9.2, SAS Institute Inc., Cary, NC, USA). Unless stated otherwise, data are presented as mean ± standard deviation (SD). Statistical significance was concluded with two-tailed *P* values less than 0.05. The experimental unit was each individual dog.

#### Primary outcome variable

The primary outcome variable was treatment failure, with superiority established by a statistically significant lower proportion of failures in the robenacoxib group compared to the placebo group. A random effects generalized linear mixed model was utilized (SAS PROC GLIMMIX) with ‘treatment’ as a fixed effect and ‘site’ and ‘treatment by site’ as random effects. The analysis involved a binary response; therefore a binomial distribution with a logit link was utilized. The covariance was modeled using the variance components structure. All sites had multiple evaluable subjects in each treatment group (at least 8 evaluable cases per site) and were therefore included in the primary efficacy analysis.

In addition, the ‘time to rescue therapy’ for each dog was assessed via a Kaplan-Meier time to event plot with comparison of groups using the log-rank, generalized Wilcoxon and likelihood ratio tests (SAS PROC LIFETEST).

#### Secondary outcome variables

A total pain score was calculated for each animal at each time point as the sum of the pain category scores at that time where total pain score = vocalization + attention to wound area (surgical site) + mobility + response to touch + demeanor + posture/activity scores.

The last observation carried forward (LOCF) method was applied to the data for the first 8 h after extubation for any animal that required rescue therapy on the day of surgery. Repeated measures analysis of covariance (RMANCOVA; SAS PROC MIXED) was utilized with ‘treatment’, ‘time’ and ‘treatment x time’ as fixed effects, and ‘site’ and ‘treatment x site’, ‘site x time’, and ‘treatment x site x time’ as random effects. The pre-treatment total pain score was included in the model as a fixed covariate. Models incorporating the covariance structures Compound Symmetry and Heterogeneous Compound Symmetry were explored, with the structure yielding the lower Akaike Information Criterion selected for the final analysis.

The individual component variables contributing to the total pain score were also analyzed using LOCF data from the day of surgery (extubation to hour 8) and the same statistical model described for the total pain score analysis.

The Shapiro Wilks test (SAS PROC UNIVARIATE) was used to assess normality of the residuals from the linear mixed models for total pain score and each of the contributing component variables. Where non-normality was observed, the non-parametric Wilcoxon Rank Sum test was used to compare the groups.

Body weight was evaluated statistically using analysis of covariance (ANCOVA; SAS PROC MIXED) with the pre-treatment body weight used as a covariate. The model included the fixed effect of ‘treatment’. In addition, summary statistics for body weight at baseline and at study exit, and the difference between the study exit and baseline body weights, were calculated for each group.

Hematology, serum chemistry, and urinalysis variables were evaluated statistically using ANCOVA (SAS PROC MIXED) with the pre-treatment value as covariate. The model included the fixed effect of ‘treatment’, with ‘site’ and the interaction ‘treatment x site’ as random effects.

The incidence of AEs in the two groups was compared with Fisher’s Exact test (SAS PROC FREQ).

## Results

### Study dogs and drugs administered

A total of 239 client-owned dogs were enrolled in the study (119 dogs received robenacoxib and 120 received placebo) and included in the demographic and safety analyses. Efficacy analyses were performed on 231 animals (116 in the robenacoxib group and 115 in the placebo group). The number of cases enrolled per investigational site for safety (efficacy) analyses was 1(0), 10 (9), 10 (10), 11 (11), 20 (19), 20 (20), 24 (22), 28 (28), 30 (28), 41 (41) and 44 (43). Eight cases were excluded from the efficacy analyses due to the use of forbidden concomitant treatments (*n* = 2), staff-owned pet (*n* = 2), surgery complications leading to death/euthanasia (*n* = 2), inaccurate dosing (*n* = 1) and insufficient number of enrolled cases at site (*n* = 1).

Demographic and baseline variables are shown in Table 2. Differences between groups were not significant, and it was concluded that the randomization had effectively created balanced groups.

**Table 2:** Demographic, breed and surgery variables; data are mean (± SD) or number of dogs (%)

Variable	Robenacoxib	Placebo	Total	*P* value*
Number of dogs	119 (49.8%)	120 (50.2%)	239 (100.0%)	
Age (years)	6.2 (±4.3)	5.5 (±3.8)	5.8 (±4.0)	0.23
Body weight (kg), pre-enrollment	20.0 (±12.2)	22.9 (±15.1)	21.5 (±13.8)	0.10
Sex and neutered status				0.068
Female intact	40 (33.6%)	33 (27.5%)	73 (30.5%)	
Female spayed	36 (30.3%)	34 (28.3%)	70 (29.3%)	
Male intact	7 (5.9%)	20 (16.7%)	27 (11.3%)	
Male castrated	36 (30.3%)	33 (27.5%)	69 (28.9%)	
Breed				0.65
Labrador Retriever	11 (9.2%)	14 (11.7%)	25 (10.5%)	
Golden Retriever	10 (8.4%)	5 (4.2%)	15 (6.3%)	
Mix-Labrador Retriever	5 (4.2%)	7 (5.8%)	12 (5.0%)	
Shih Tzu	6 (5.0%)	5 (4.2%)	11 (4.6%)	
Various other breeds	87 (73.1%)	89 (74.2%)	176 (73.6%)	
Type of surgery				0.62
Skin tumor removal (≥ 8 cm in size)	31 (26.1%)	39 (32.5%)	70 (29.3%)	
Ovariohysterectomy	29 (24.4%)	26 (21.7%)	55 (23.0%)	
Cystotomy	18 (15.1%)	17 (14.2%)	35 (14.6%)	
Gastropexy	12 (10.1%)	16 (13.3%)	28 (11.7%)	
Other soft tissue surgery	29 (24.4%)	22 (18.3%)	51 (21.3%)	

*Significance of differences between treatment groups (based on t-test for continuous variables and χ^2^ test for categorical variables)

The mean (range) ages of dogs were 6.2 years (6 months to 14 years) in the robenacoxib group and 5.5 years (6 months to 16 years) in the placebo group. The weight range at pre-treatment was 2.7–55.0 kg in the robenacoxib group and 3.2–63.7 kg in the placebo group. There were more female than male dogs in the study.

The most common breeds were Labrador Retriever, Golden Retriever, Mix-Labrador Retriever and Shih Tzu. The predominant soft tissue surgeries in both treatment groups were skin tumor removal of mass ≥ 8 cm in size (*n* = 70), ovariohysterectomy (*n* = 55), cystotomy (*n* = 35) and gastropexy (*n* = 28).

All cases received butorphanol as a pre-operative medication. Propofol was used as the anesthesia medication for induction; isoflurane and sevoflurane were used as the anesthesia mediations for maintenance. Antibiotics were administered to 55.7% of dogs at the time of surgery. The most frequently used concomitant treatments were analgesics, fluids and antibacterials.

### Primary outcome variable

During the study, a total of 163 dogs were considered treatment success with 89 of 116 cases (76.72%) in the robenacoxib group compared to 74 of 115 cases (64.35%) in the placebo group. The percentage of treatment failure was therefore 23.28% with robenacoxib and 35.65% with placebo. There was a significant difference in the proportion of success/failures in the robenacoxib group compared to the placebo group (*P* = 0.019) (Table 3). All treatment failures were due to administration of rescue therapy with CMPS-SF scores ≥6; no dogs received rescue therapy with a CMPS-SF <6 and none were classified as a treatment failure due an AE.

**Table 3:** Primary outcome variable: the frequency of success and failure outcomes by group

Group	Outcome		Total	*P* value
	Success	Failure (Withdrawn from study)		
Robenacoxib	89 (76.72%)	27 (23.28%)	116	0.019
Placebo	74 (64.35%)	41 (35.65%)	115	
Total	163	68	231	

**Fig. 1:**
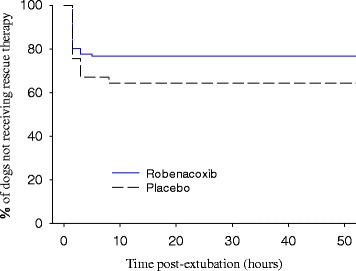
Kaplan-Meier plot of time to rescue analgesia therapy

The Kaplan-Meier plot of ‘time to rescue analgesia therapy’ is presented in Fig. 1. All rescues occurred at or before 8 h post-extubation, with 51/68 (75.0%) at ≤1.5 h, 64/68 (94.1%) at ≤3 h and 65/68 (95.6%) at ≤5 h. The number of dogs receiving rescue therapy at the 1.5, 3, 5 and 8 h time points (or in the interval since the previous time point) was respectively 23, 3, 1 and 0 in the robenacoxib group (total 27) and 28, 10, 0 and 3 in the placebo group (total 41).

In the time to event analysis, the log-rank and likelihood ratio tests were both statistically significant (*P* = 0.046 and *P =* 0.016) in favor of the robenacoxib group, while the generalized Wilcoxon test also provided some evidence of a favorable effect of robenacoxib (*P* = 0.072). The robenacoxib group had a lower probability of failures (rescues) beginning at 1.5 h post-extubation and at all subsequent remaining time periods compared to the placebo group.

### Secondary outcome variables

**Fig. 2:**
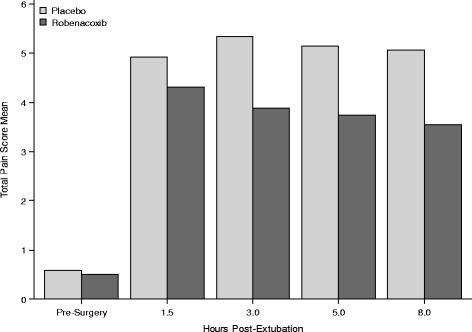
Mean total pain score

The least squares mean (LSMean) total pain scores showed statistically significant differences between groups and in favor of robenacoxib (experiencing less pain) at 3 and 5 h (*P* < 0.05) and 8 h post-extubation (*P* < 0.01) (Fig. 2, Table 4), including the mean total pain score (with LOCF) over time between both groups. Analyses for the six individual components contributing to the total pain score using analogous models were conducted, again utilizing LOCF through the first 8 h after extubation (Table 4). Pain at the surgery sites (response to touch) was significantly improved at 3, 5 and 8 h post-extubation in dogs receiving robenacoxib versus placebo (*P* < 0.01). Furthermore, a significant overall improvement in posture/activity was revealed with robenacoxib having lower scores versus placebo (*P* < 0.01).

**Table 4:** Secondary outcome variables

Variable	Time Point (Day: Time)	Robenacoxib LSMean	Placebo LSMean	LSMean Difference	*P* value (RMANCOVA)	*P* value (Wilcoxon Rank Sum)
Total Pain Score	0: 1.5 h	3.89	4.53	−0.648	0.27	0.558
	0: 3 h	3.44	4.95	−1.51	0.011*	0.003**
	0: 5 h	3.32	4.76	−1.44	0.015*	0.008**
	0: 8 h	3.12	4.69	−1.56	0.008**	0.014*
Vocalization	Overall^a^	0.252	0.309	−0.057	0.55	0.482
Attention to Wound/Surgical Site	Overall^a^	0.166	0.208	−0.042	0.47	0.435
Mobility	0: 1.5 h	0.960	0.867	0.093	0.57	0.276
	0: 3 h	0.745	0.945	−0.200	0.18	0.392
	0: 5 h	0.668	0.936	−0.268	0.072	0.170
	0: 8 h	0.607	0.884	−0.276	0.058	0.132
Response to Touch	0: 1.5 h	0.878	1.22	−0.342	0.098	0.128
	0: 3 h	0.846	1.46	−0.612	0.003**	0.001**
	0: 5 h	0.843	1.45	−0.612	0.003**	0.002**
	0: 8 h	0.796	1.48	−0.682	0.001**	0.001**
Demeanor	Overall^a^	0.995	1.13	−0.138	0.17	0.251
Posture/Activity	Overall^b^	0.463	0.816	−0.353	0.006**	0.028*

The Last Observation Carried Forward (LOCF) method was used (see Methods); *h* hour, *LSMean* Least Squares Mean

*LSMean* difference = *LSMean* of the robenacoxib group - *LSMean* of the placebo group, *RMANCOVA *Repeated Measures Analysis Of Covariance

^a^Treatment x time interaction was not statistically significant (*P* > 0.05). Therefore, results for the main effect of treatment are presented. The Wilcoxon rank sum test was performed on the median scores for each component obtained for each dog over the period 1.5 h to 8.0 h post-extubation

^b^Treatment x time interaction was not statistically significant (*P* = 0.11). However, a statistically significant overall effect of treatment was observed (*P* = 0.006 via RMANCOVA; *P* = 0.028 via the Wilcoxon rank sum test)

*Statistically significant at *P* < 0.05

**Statistically significant at *P* < 0.01

Significant non-normality (*P* < 0.01) was detected in the residuals from the linear mixed models for total pain score and each of the component variables contributing to the total score. Re-analysis for each of these variables using the Wilcoxon Rank Sum test produced similar results to those obtained via the generalized linear model (Table 4).

### Other observations

The percentage of dogs receiving rescue therapy by the predominant surgery types skin tumor removal, ovariohysterectomy, cystotomy and gastropexy was respectively 22.6%, 20.7%, 11.1% and 30.8% in the robenacoxib group and 21.1%, 39.1%, 37.5% and 44.4% in the placebo group.

### Safety - adverse events

A summary of the number and percentage of cases with each reported AE is depicted in Table 5. The frequency of dogs with recorded AEs did not differ significantly between the two groups (*P* = 0.100). Gastrointestinal tract disorders (particularly diarrhea and vomiting) were the most commonly observed AEs in both groups. Three serious AEs were reported (one case in the robenacoxib group and two cases in the placebo group). One dog treated with robenacoxib died due to circulatory collapse after surgical resection of a chronic intestinal blockage with extensive tissue devitalization. One placebo-treated dog was euthanized due to diagnosis of advanced neoplasia during surgery. These two cases were considered unrelated to treatment. Finally, one dog in the placebo group had dramatically elevated liver enzymes at study exit; follow up blood work showed these returned to values within or close to normal ranges.

**Table 5:** Adverse events reported during the study

Adverse event^a^	Robenacoxib		Placebo		*P* value
	*N*	% of total (*n* = 119)	*N*	% of total (*n* = 120)	
Vomiting	6	5.0%	4	3.3%	0.544
Diarrhea	6	5.0%	3	2.5%	0.330
Decreased appetite	3	2.5%	0	0%	0.122
Weight loss	1	0.84%	0	0%	0.505
Hypotension	1	0.84%	0	0%	0.505

*P* values were calculated with the Fisher’s Exact test

^a^Dogs may have experienced more than one type or occurrences of an event

A total of 10 post-intervention AEs were experienced in 2 dogs in the robenacoxib group and in 6 dogs in the placebo group. In the robenacoxib group, one dog had diarrhea, while vomiting was reported in a second dog. In the placebo group, two dogs vomited, two dogs had elevated liver enzymes, one dog showed signs of redness and swelling at the surgical site, and for one dog hematoma at the dorsal aspect of the incision was reported with grade II lameness of the left hind limb and painful general appearance.

Based on results from follow-up phone calls, owners reported abnormal findings in 29 dogs (12 dogs that received robenacoxib and 17 dogs that received placebo). The majority of signs reported were consistent with those associated with surgery (e.g. local swelling at surgical site, skin and tissue infection or inflammation) or digestive tract disorders (e.g. vomiting and diarrhea).

### Safety - clinical pathology

Summary data for selected hepatic, hematologic and renal variables are shown in Table 6. Mean values for all hepatic and hematology variables at pre-treatment and study exit were within the normal reference ranges in both groups. There were no significant differences between the robenacoxib and placebo groups.

**Table 6:** Selected hepatic, hematologic and renal variables at pre-treatment and study exit (mean ± SD)

Variable (Laboratory reference range)	Time	Robenacoxib (*n* = 118)			Placebo (*n* = 119)			*P* value
		Mean (±SD)	Cases^a^		Mean (±SD)	Cases^a^		
			High	Low		High	Low	
Serum								
Urea nitrogen, mg/dL (6–31 mg/dL)	Pre-Treatment	18.1 (±7.3)	5	0	18.3 (±6.5)	5	0	0.078
	Study Exit	14.9 (±4.8)	1	0	13.6 (±4.1)	0	0	
Creatinine, mg/dL (0.5–1.6 mg/dL)	Pre-Treatment	0.91 (±0.25)	2	1	0.96 (±0.28)	2	2	0.763
	Study Exit	0.79 (±0.21)	0	5	0.81 (±0.20)	0	1	
Alkaline phosphatase, U/L (5–131 U/L)	Pre-Treatment	141.8 (±239.7)	26	0	112.0 (±214.7)	23	0	0.233
	Study Exit	162.1 (±281.5)	30	0	116.4 (±176.3)	21	0	
Alanine aminotransferase, U/L (12–118 U/L)	Pre-Treatment	55.3 (±41.6)	6	0	54.0 (±50.0)	7	1	0.613
	Study Exit	53.7 (±49.4)	5	0	59.1 (±101.2)	5	1	
Aspartate aminotransferase, U/L (15–66 U/L)	Pre-Treatment	29.6 (±10.5)	1	1	28.8 (±7.8)	0	1	0.449
	Study Exit	37.5 (±46.6)	6	1	41.7 (±41.3)	11	0	
Total bilirubin, mg/dL (0.1–0.3 mg/dL)	Pre-Treatment	0.14 (±0.06)	1	0	0.15 (±0.05)	0	0	0.431
	Study Exit	0.15 (±0.07)	3	0	0.15 (±0.07)	2	0	
Total protein, g/dL (5.0–7.4 g/dL)	Pre-Treatment	6.6 (±0.54)	7	0	6.5 (±0.57)	7	0	0.806
	Study Exit	6.4 (±0.53)	4	0	6.4 (±0.57)	6	0	
Albumin, g/dL (2.7–4.4 g/dL)	Pre-Treatment	3.6 (±0.34)	0	1	3.6 (±0.34)	0	1	0.501
	Study Exit	3.5 (±0.32)	0	1	3.4 (±0.36)	0	2	
Hematology								
Hemoglobin, g/dL (12.1–20.3 g/dL)	Pre-Treatment	16.2 (±2.1)	2	3	16.4 (±1.8)	0	1	0.380
	Study Exit	15.1 (±1.8)	0	8	15.0 (±1.8)	0	8	
Hematocrit, % (36–60%)	Pre-Treatment	49.8 (±6.4)	5	1	50.2 (±5.5)	2	0	0.302
	Study Exit	46.5 (±5.2)	0	5	46.2 (±4.9)	1	1	
Red blood cell count, 10^12^/L (4.8–9.3 10^12^/L)	Pre-Treatment	6.8 (±0.89)	0	0	6.9 (±0.76)	0	0	0.406
	Study Exit	6.3 (±0.78)	0	2	6.4 (±0.70)	0	0	
White blood cell count, 10^9^/L (4.0–15.5 10^9^/L)	Pre-Treatment	10.9 (±3.7)	13	0	10.4 (±3.0)	6	1	0.454
	Study Exit	13.5 (±4.0)	32	0	13.6 (±4.4)	37	0	
Urine								
Urine specific gravity (1.015–1.050)	Pre-Treatment	1.03 (±0.02)	23	8	1.03 (±0.01)	13	15	0.839
	Study Exit	1.04 (±0.02)	27	5	1.04 (±0.02)	25	5	

*P* values were obtained from an analysis of covariance of the study exit values for each variable, with the pre-treatment value for each variable used as a covariate. Only cases for which both pre-treatment and study exit results were available were included in the analysis

^a^Number of cases with values higher and lower than the reference range pre-treatment and at study exit

In both groups, mean values for all serum chemistry and urine variables were within normal reference ranges at pre-treatment and study exit, with the exception of blood urea nitrogen (BUN)/creatinine ratio (higher in the robenacoxib group, *P* = 0.035) and urine pH (higher in the placebo group, *P* = 0.018). The LSMean difference in BUN/creatinine ratio for both groups at study exit was 2.23 (19.70 for robenacoxib versus 17.47 for placebo). The LSM difference for urine pH at study exit was −0.24 (6.52 for robenacoxib versus 6.76 for placebo).

### Safety - body weight change

The change in body weight was similar between groups (i.e. body weight at study exit minus body weight at baseline). There were no significant (*P* = 0.512) differences in mean study exit body weight between the robenacoxib and placebo groups after adjusting for pre-enrollment body weight.

## Discussion

Robenacoxib tablets, administered orally at a dose of 2 mg/kg (an inherent dose band of 2–4 mg/kg) prior to soft tissue surgery and again once daily for up to two days after surgery, provided superior analgesic effects when compared to a placebo as measured by the need for rescue therapy. There was a statistically significant reduction in the proportion of dogs requiring rescue therapy (*P* = 0.019) in favor of robenacoxib (23.28%) compared to placebo (35.65%). Furthermore, oral robenacoxib administration was well tolerated.

The study was designed as a prospective, multi-center, randomized, masked, placebo-controlled, parallel-group clinical trial. The study was adequately powered as superiority of robenacoxib versus placebo was statistically proven for the primary outcome variable with a balanced 1:1 ratio of cases between the two treatment groups.

All surgical procedures were performed by experienced surgeons from 11 clinics at different locations in the USA. Dogs enrolled were of various breeds and the types of soft tissue surgery performed are representative of cases in general practice. More female dogs were enrolled than male dogs due to the inclusion and exclusion criteria, i.e., including ovariohysterectomy but not castration (the later surgery type considered to be less painful) [1]. Efforts were taken to minimize the pain potentially suffered by dogs receiving placebo. All dogs were hospitalized and received butorphanol as pre-anesthetic medication pre-operatively, there were relatively frequent obligatory observation time points and rescue (pain) therapy could be given any time at the veterinarians' discretion.

It is generally accepted that surgery-associated pain is most severe in the early post-operative period [2, 3], and then decreases with time as inflammation subsides and healing occurs. In several studies the duration of post-operative assessments did not proceed beyond 24 h after surgery [17–21] or was considerably shorter, lasting only a few hours after tracheal extubation [22]. Furthermore, it is recommended to start analgesic therapy before the potentially painful event rather than attempt to regain control of pain after it occurs [23–27]. Depending on the surgery type, the perception of pain can be different [1]. Interestingly, in our study the percentage of dogs requiring rescue therapy was highest after gastropexy in both groups indicating that pain was perceived to be more severe for this surgery type.

Behavioral assessments were performed as accurately as manageable under practical field conditions, and pain assessments were made using the CMPS-SF. Dogs were hospitalized throughout the study and kept in a quiet location. The assessments for the primary outcome variable were based on the opinion of the clinician and not of the owner. To ensure interpretative consistency for pain assessments, the person selected to evaluate the efficacy variables was trained and was the same individual for each assessment from the Day 0 clinical baseline to study exit for a given animal. The CMPS-SF was selected as it is a validated composite scale for assessing acute pain in dogs in a hospital setting based on observations and behaviors [2, 16, 21, 28]. In addition, the CMPS-SF provides guidance with regard to need for analgesia i.e. rescue therapy is required if the total score is 6 or greater. In this study, no case was judged by the Investigators to require rescue therapy with a CMPS-SF < 6, although it was permitted at any time at the discretion of the Investigators.

A limitation of the study was that analyses of the secondary outcome variables were challenging due to the unequal frequency of withdrawal of cases after administration of rescue therapy between the two groups, with the placebo group having more rescues within the first 8 h than those dogs treated with robenacoxib. The data were therefore analyzed using the LOCF method. The LOCF method [29] has limitations, but was justified in this study since it was used for cases proactively withdrawn due to lack of efficacy and for a limited period (up to 8 h post-extubation).

Robenacoxib, like other NSAIDs, decreases inflammation, but direct measurement of inflammation is generally not possible in clinical studies as the principle site of inflammation is frequently not visible. Inflammation was evaluated indirectly in our study as part of the CMPS-SF. Robenacoxib has been shown to have anti-inflammatory properties, evidenced from inhibition of swelling in model studies in rodents [10] and dogs [5, 6].

The efficacy of various NSAIDs in peri-operative pain management has been investigated previously in clinical studies in dogs [11, 12, 25, 26]. Comparison of results between studies is difficult due to different study designs, anesthetic procedures, concomitant drugs, NSAID treatment durations, types of surgeries as well as pain assessment methods. In a previous study, the efficacy of robenacoxib and meloxicam was investigated in dogs undergoing soft tissue surgery [12]. The efficacy of robenacoxib was at least as good (i.e. statistically non-inferior) to the positive control, meloxicam. In that study, pain and inflammation were assessed subjectively by clinicians using the Glasgow Composite Pain Scale (GCPS) [30]; unweighted results were reported since weighting factors for the indices had not been published at the time the study was initiated [21, 28]. There were no specific criteria defined when rescue therapy should be used and no dog received such therapy. The effect of two other NSAIDs, administered as a single oral dose pre-surgery followed by 2 days post-surgery, for the control of pain and inflammation in soft tissue surgery in dogs was investigated in the following two studies. For firocoxib, superior efficacy versus a negative control was reported with a comparable design to our study and pain assessment using the CMPS-SF [26]. The frequency of rescue therapy was 16.4% for the active and 50.0% for the negative control. For deracoxib, superior efficacy versus a placebo control was reported. The frequency of rescue therapy was 12.5% (2/16) compared to placebo 56.3% (9/16) [25]. However in that study, pain was assessed using a different method (GCPS). The incidence of rescue therapy (range 12.5 to 23.28%) in clinical trials testing NSAIDs in dogs undergoing soft tissue surgery indicates that pain is a complex phenomenon which may differ between individuals [3] and, although NSAIDs provide an important contribution to pain management, it is not surprising that NSAIDs alone may not produce optimal results. It is well established that optimal peri-operative pain management regimens should incorporate drugs from several classes i.e. multi-modal therapy [1, 3].

Reported AEs, clinical pathology variables and results of clinical examinations by the Investigators indicated that oral administration of robenacoxib was well tolerated. The most frequently reported AEs were gastrointestinal tract disorders such as vomiting and diarrhea.

Robenacoxib has a good safety index in healthy dogs, producing no biologically relevant toxicity at oral doses as high as 40 mg/kg daily for 1 month and up to 10 mg/kg daily for 6 months [8]. There was no evidence from this study of any toxicity of robenacoxib to target organs that are most sensitive to NSAID toxicity (gastrointestinal tract, kidney and liver). Although some statistically significant differences in clinical chemistry (BUN/creatinine ratio was higher in the robenacoxib group) and urinalysis (pH was higher in the placebo group) were found between groups, the means of all these variables remained within normal clinical ranges at the end of the study and findings were not considered to be clinically relevant.

## Conclusions

Robenacoxib by oral (tablet) administration at a target dose of 2 mg/kg (an inherent dose band of 2–4 mg/kg) prior to soft tissue surgery and again once daily for up to two days after surgery was effective and well tolerated in the control of peri-operative pain and inflammation in dogs undergoing soft tissue surgeries.

## Appendix

**Table:** The short form of the Glasgow Composite Measure Pain Scale (CMPS-SF) [16] used by the clinicians to assess the dogs

Circumstance	Assessment and Scale
Look at the dog in kennel	Vocalization: Is the dog: [0] quiet [1] crying or whimpering [2] groaning [3] screaming Attention to wound area: Is the dog: [0] ignoring any wound or painful area [1] looking at wound or painful area [2] licking wound or painful area [3] rubbing wound or painful area [4] chewing wound or painful area
Dog out of kennel on lead	Mobility: When the dog rises/walks is it: [0] normal [1] lame [2] slow or reluctant [3] stiff [4] it refuses to move
Response to touch	Response to touch: Does the dog: [0] do nothing [1] look around [2] flinch [3] growl or guard area [4] snap [5] cry
Overall assessment	Demeanor: Is the dog: [0] happy and content or happy and bouncy [1] quiet [2] indifferent or non-responsive to surroundings [3] nervous or anxious or fearful [4] depressed or non-responsive to stimulation Posture: Is the dog: [0] comfortable [1] unsettled [2] restless [3] hunched or tense [4] rigid

## Abbreviations

AE: Adverse Event
ANCOVA: Analysis Of Covariance
BUN: Blood Urea Nitrogen
CMPS-SF: Glasgow Composite Measure Pain Scale - Short Form
COX: Cyclooxygenase
GCPS: Glasgow Composite Pain Scale
LOCF: Last Observation Carried Forward
LSMean: Least Squares Mean
NSAID: Non-Steroidal Anti-Inflammatory Drug
RMANCOVA: Repeated Measures Analysis Of Covariance
SD: Standard Deviation
